# Exploring nursing faculty, managers, newly graduated nurses, and students’ experiences of nursing internship program implementation in Iran: a descriptive qualitative study

**DOI:** 10.1186/s12912-022-01159-8

**Published:** 2022-12-27

**Authors:** Amir Shahzeydi, Fariba Taleghani, Maryam Moghimian, Sedigheh Farzi, Ahmadreza Yazdannik, Kolsoum Farzi

**Affiliations:** 1 Isfahan University of Medical Sciences, Isfahan, Iran; 2 Nursing and Midwifery Care Research Centre, Department of Adult Health Nursing, Faculty of Nursing and Midwifery, Isfahan University of Medical Sciences, Isfahan, Iran; 3 Nursing and Midwifery Sciences Development Research Center, Najafabad Branch, Islamic Azad University, Najafabad, Iran; 4 Nursing and Midwifery Care Research Center, Department of Adult Health Nursing, Faculty of Nursing and Midwifery, Isfahan University of Medical Sciences, Isfahan, Iran; 5 Nursing and Midwifery Care Research Center, Department of Critical Care Nursing, Faculty of Nursing and Midwifery, Isfahan University of Medical Sciences, Isfahan, Iran; 6 Lorestan University of Medical Sciences, Khorramabad, Iran

**Keywords:** Internship, Nursing, Student, Faculty, Manager

## Abstract

**Background:**

Nursing students are required to acquire the necessary clinical knowledge and skills to provide safe and quality care. The method of providing training, particularly for final-year nursing students, is of utmost importance. An internship is a program during which students work in shifts similar to nurses employed in a hospital; however, the number of their shifts and patients is less than nurses; a nurse and the faculty supervise the care they provide, and they are paid a monthly salary. This study was conducted to explore nursing faculty, managers, new graduates, and students’ experiences of nursing internship program implementation.

**Methods:**

This descriptive qualitative study was conducted from November 2021 to March 2022. The participants were selected from among nursing managers, newly graduated nurses, nursing internship students (final-year undergraduate), and faculty of Iran. Data were collected using in-depth semi-structured interviews. The qualitative content analysis approach was used for data analysis.

**Results:**

Participants in this study included 17 nursing internship students, 12 nursing managers, three faculty members, ten nursing preceptors, and five newly graduated nurses from the internship program, 47 participants in total. After analyzing the data, five themes, including ‘facilitation of socialization process,’ ‘filling the gap between theory and practice,’ ‘improving self-confidence and independence,’ ‘an opportunity for clinical skill training,’ and ‘Achilles’ heel of the clinical setting,’ and nineteen subthemes were extracted from the participants’ experiences.

**Conclusion:**

Implementation of an internship program for final-year nursing students plays a role in preparing them for better professional performance, enhancing clinical skills, increasing self-confidence and independence, inspiring the nursing profession, strengthening professional commitment, and improving the chances of employment after graduation. In order to alleviate the identified challenges of the internship program, holding a briefing meeting with managers, supervisors, and faculty to determine working hours, performance standards, and amenities such as lunch, dinner, and resting place is efficient.

## Background

Nursing students are required to acquire the necessary clinical knowledge and skills to provide safe and quality care [1–3]; consequently, the method of providing training, particularly for final-year nursing students, is of utmost importance [4]. An internship is a program during which students work in shifts similar to nurses employed in a hospital; however, the number of their shifts and patients is less than nurses; a nurse and the faculty supervise the care they provide, and they are paid a monthly salary [5, 6]. Internship programs provide a context in which students can put what they have learned into practice [7]. The internship program began in the United States in 1900 [8] and provided the necessary platform in nursing schools to transition from a nursing student to a new graduate [9]. This educational model is held for novice nurses in some countries and final-year nursing students in others [5, 10].

An internship provides a worthwhile learning experience before entering the nursing profession and allows students to professionally apply the principles and theories learned in school. The internship program also has the potential to increase students’ self-confidence, critical thinking, knowledge, skills, and clinical competence and reduce their stress [11, 12]. During this program, students are offered the opportunity to explore the profession, develop their personalities, and acquire new skills [8]. From an organizational perspective, the internship program allows organizations to produce more experienced graduates. From the students’ viewpoint, the program provides a platform for the development of clinical skills that may not be taught in classrooms [13].

Studies show that novice nurses who have not experienced an internship program lack sufficient self-confidence and the ability to provide efficient and quality care in clinical settings upon entering the clinical environment [14]. In Abdelkader et al.’s study (2020), nursing students were gratified with the internship courses conducted and considered it essential to improve their clinical practice [15]. Likewise, in the study by Hardie et al. (2018), it was found that internship students acquired teamwork, communication, interpersonal, and dilemma management skills [13].

Despite the benefits of the internship course, the program faces challenges such as inadequate faculty support, the faculty and hospital’s poor collaboration, the hospital’s unusual and improper expectations of students, the faculty’s insufficient academic and practical competence to supervise, and the faculty’s inadequate time allocated to students. Moreover, nurses have to work with students despite their heavy workload, inadequate staff, and the collaboration of a small number of or no faculty members. Under these circumstances, they are unable to effectively manage nursing students. In addition, students ought to be considered a vulnerable group since complete separation from the school and faculty is stressful for them, and there is a risk of exploiting students as free or cheap labor by hospitals [12].

The internship program in Iran began for the first time at Isfahan University of Medical Sciences in 2018 and has continued to date. In this program, final-year nursing students fulfill their internship program in various hospital wards, including 20 shifts (morning, evening, and night) per month, and are paid a monthly salary. At the time of patient assignment, the head nurses assign two patients to each student in the first week, three in the second, and four in the third and fourth weeks. Students are independently responsible for patients; however, they work under the nurse’s supervision and raise questions and problems. Besides, resident faculty visit students at least four times a month, evaluate their knowledge and skill, and provide feedback on their problems. Students ask professors about their problems during the visit sessions and share their problems with the ward and the entire course. It should be noted that before the start of the internship program, essential nursing concepts such as patient safety, nursing process, medication calculations, and nursing documentation are retaught to students.

In order to improve, a new curriculum is required to be evaluated from stakeholders’ perspectives to identify benefits and gaps [16]. Qualitative research provides researchers with the opportunity to discover and explain the realities of the clinical setting and further understand the challenges. Therefore, this study was conducted to explore nursing faculty, managers, newly graduated nurses, and students’ experiences of nursing internship program implementation.

## Methods

### Study design

This descriptive qualitative study was conducted from November 2021 to March 2022. In this study type, the researcher provides a comprehensive summary of the phenomenon or related events in a common language but does not go into the deep phase of interpretation [17]. These studies are less interpretive than other qualitative methods, such as phenomenological or grounded theory research [18].

### Setting and sample

The participants were selected from among faculty, nursing managers, nursing internship students (final-year undergraduates), and newly graduated nurses from six hospitals in Iran. The inclusion criteria for nursing internship students included at least three months of experience as an internship student in the clinical setting(Medical/Surgical, Intensive Care Units(ICU), Cardiac Care Unit(CCU), pediatric wards) and willingness to participate in the study.

Inclusion criteria for faculty, newly graduated nurses, and nursing managers included at least one year of teaching and supervising experience of nursing internship students and willingness to participate in the study. They were excluded from the study if they refused to share their experiences. Participants were selected using a purposeful sampling method. Sampling was performed with maximum variation in terms of age, gender, and the number of years of work, teaching, and supervising experience.

### Data collection

After coordinating with the educational manager of the school, contact numbers of the nursing internship student (at least three months of experience as an internship student in the clinical setting), faculty (at least one year of supervising experience), nursing managers of hospitals, and newly graduated nurses were obtained. We contacted participants, and If they were willing to participate in the study, the interview time and place were arranged at their convenience. Data were collected from November 2021 to March 2022 using in-depth semi-structured interviews. All interviews were conducted by the corresponding author (SF), who has Ph.D. in nursing and has published several qualitative articles. The interviews lasted for 30–45 min on average, and they all began with a general question to establish a close relationship with participants (Table 1). Participant selection and sampling continued until data saturation was reached. Saturation refers to the repetition of discovered information and confirmation of previously collected data [19]; when ongoing analysis reveals no new information and no new categories emerge, sampling may cease [20].

**Table 1:** Samples of interview questions

Participants	Questions
Students and newly graduated nurses	What do you think of the internship program? Please explain your positive and negative experiences of an internship program. How would you plan for the internship program if you were in charge of it?
Faculty	What positive and negative experiences have you had or observed in students during student supervision? What do you think are the advantages and disadvantages of this program?
Nursing managers	What do you think about the internship program? What is the difference between the performance of internship graduates and others?

### Data analysis

The qualitative content analysis approach proposed by Graneheim and Lundman was used for data analysis [21]. Recorded interviews were transcribed verbatim and read by the researchers several times. Following that, meaning units were extracted. Identified meaning units were condensed, abstracted, and coded. Finally, similar codes were grouped under subthemes, and themes were formed using the inductive process.

### Trustworthiness

Rigor was ensured using the confirmability, credibility, dependability, and transferability criteria [22]. We enhanced confirmability by bracketing and maintaining a clear, easy-to-follow audit trail of all research activities and analysis notes. To strengthen credibility, we conducted peer debriefing and reviewed the data, codes, subthemes, and themes. The extracted codes and results were retrieved and shared with the participants to validate the congruency of the codes with their experiences. Dependability was achieved through the participation of more than one researcher in data analysis. Selecting participants with different demographic characteristics enhanced the transferability of the results.

## Results

Participants in this study consisted of 17 nursing internship students, 12 nursing managers, three faculty members, ten nursing preceptors, and five newly graduated nurses from the internship program. The demographic characteristics are presented in Table 2. After analyzing the data, five themes and nineteen subthemes were extracted from the participants’ experiences (Table 3).

**Table 2:** Participants’ characteristics

Variables		Mean ± SD or N (%)
Participant	Nursing internship students	17 (36.2%)
	Nursing managers	22( 46.8%)
	Faculty	3 (6.4%)
	Nurses newly graduated from the internship program	5 (10.6%)
Gender	Male	19(40.4%)
	Female	28 (59.6)
Level of education	undergraduate	17( 36.2%)
	Bachelor	22( 46.8%)
	Master	6 (12.7%)
	Ph.D.	2 (4.3%)
Age	34.80 ± 12.28	
Work experience (year)	11.25 ± 10.47	

**Table 3:** The themes and subthemes

Themes	Subthemes
Facilitation of the socialization process	Role model nurses Awareness of and adherence to professional rules and regulations Gaining a professional identity Awareness of different roles in nursing
Filling the gap between theory and practice	The presence of the faculty in the ward as a clinical supervisor Transferring nurses’ clinical experiences to internship nursing students Participation of internship students and clinical supervisors in nurse education Nursing managers’ willingness to recruit nursing internship students after graduation
Improving self-confidence and independence	Being approved by nurses Providing direct patient care An internship student as a member of the health care team.
Opportunity for clinical skill training	Increasing the frequency of nursing procedures Diverse nursing procedures in different shifts Exposure to a wide range of patients
Achilles’ heel of the clinical setting	A risk of routine-centeredness instead of patient-centeredness Delegated clinical education Imposing more clinical work on the student Lack of amenities

### Facilitation of the socialization process

This theme includes four subthemes: “role model nurses,” “awareness of and adherence to professional rules and regulations,” “gaining a professional identity,” and “awareness of different roles in nursing.” Participants’ experiences showed that students in the internship program chose nurses, especially those with high knowledge and clinical competence, as their role models. Students prepare for the transition to nursing as they provide professional care and learn about providing safe and quality care. One of the students stated: “We worked in shifts work like staff, we knew how to work in different shifts; what hospital conditions are; how a nurse should work” (P_9_). Another student said in this regard: “We realized that we had to comply with a series of regulations. Someone called the head nurse, with whom we have to arrange shifts, supervises us. We have to be flexible. We learn to be accountable and understand who is responsible for the patient; what the work conditions are” (P_3_).

In addition, the participants were gratified that the internship program equipped them with the knowledge to provide high-quality nursing care to attain their patients’ trust. Nursing students experienced a transition to professional identity through gaining satisfaction with professional practice. One student said: “Now, I have learned everything, and I can introduce myself as a nurse to patients and attract their trust” (P_4_).

Internship students are also introduced to nurse roles in different wards and prepare to take various nurse roles in the future. One of the faculty members stated: “In addition to caring for the patient, the student also teaches, gets acquainted with the role of the head nurse in the management course, learns to lead a care team, responds to family caregivers’ questions, and uses acquired theoretical and practical information to educate patients and families. These activities familiarize them with different roles they will take in the future” (P_19_).

### Filling the gap between theory and practice

This theme includes four subthemes: “Presence of the faculty in the ward as a clinical supervisor,” “Transferring nurses’ clinical experiences to internship nursing students,” “Participation of internship students and clinical supervisors in nurse education,” and “Nursing managers’ willingness to recruit nursing internship students after graduation,”

Nursing internship students, faculty, and administrators claimed that the internship course could integrate theoretical nursing into practice and help train skilled nurses. One of the students stated: “The professor supervises us and answers our questions. It helps us not to do clinical practice blindly” (P_7_). Another nurse manager said: “The faculty’s supervision and responses to students’ and nurses’ questions about patient care resulted in the promoted quality of care provided by them” (P_26_). One of the nursing managers stated: “internship students’ presence, professors’ supervision, clinical rounds, and clarification of scientific ambiguities that occur at times can greatly help improve nurses’ knowledge” (P_13_).

The newly graduated nurses believed that the novice nurses who had worked as interns in a similar ward and had gained the necessary experience and skills to work in that ward were among the best nurses. One newly graduated nurse said: “The nursing internship students are much in demand due to the attendance of professors in the hospital and help to improve nursing care provided by nurses and students” (P_15_). One of the head nurses said, “I have the least trouble working with Ms. F, who used to be our internship student;, I will not allow her to be transferred to other wards” (P_1_).

### Improving self-confidence and independence

This theme includes three subthemes: “Being approved by nurses,” “Providing direct patient care,” and “An internship student; as a member of the healthcare team.” Participants’ experiences showed that an internship student is considered an auxiliary force by nurses and is assigned tasks in the care team. One of the students said, “I handled the patient in the wards like a full-fledged staff. There was no one supervising me. The staff knew we faced problems only during the first and second months. They trusted us and assigned patient care to us. Another student said, “Care was provided through the case method, and we were assigned entire patient care” (P_2_). In addition, internship nursing students gradually gained the confidence to provide direct patient care. One of the students said: “Before taking the internship course, I had a lot of information, but I wasn’t confident to practice them. I was stressed, but this course reduced my stress, and I gained confidence because the nurses accepted me into their team” (P_3_).

### Opportunity for clinical skill training

This theme can be discussed in three subthemes: “Increasing the frequency of nursing procedures,” “Diverse nursing procedures in different shifts,” and “Exposure to a wide range of patients.” Participants’ experiences showed that although nursing students became knowledgeable about various nursing procedures during the first three years of their studies, they acquired the necessary clinical competence during the internship course due to the variety of procedures and the opportunity to perform them time and again. One of the students stated, “In the previous training course, I didn’t have much opportunity for IV insertion, but when I became an internship student, I performed multiple IV insertions and became proficient” (P_16_). Another student said, “In the emergency department, there were patients of all kinds that we had never experienced in a previous training course. When I became an internship student, I learned about types of arrhythmias and strokes” (P_10_). In addition, participants’ experiences showed that the presence of internship nursing students in shifts and different wards and dealing with patients with complex problems significantly contributed to their proficiency and functional independence. One student said: “I fully learned what to do for a patient with ventricular tachycardia. In fact, I learned patient management well” (P_6_).

### Achilles’ heel of the clinical setting

This theme included four subthemes: “A risk of routine-centeredness instead of patient-centeredness,” “Delegated clinical education,” “Imposing more clinical work on the student,” and “Lack of amenities.”

The participants’ experiences showed that despite the benefits of the internship program, it would be challenged if the clinical setting was not sufficiently prepared. The student’s presence in the ward and providing care supervised by a nurse carries the risk of inappropriate role modeling. In some cases, students performed what was considered by the nurse as the ward’s routine, which did not reflect evidence-based care. In addition, students often relied on routine care and endured a great deal of stress of being negatively scored by nurses. One student said: “I felt like I was stuck in the ward routine, and I wasn’t given important information about the ward and the care system. Although I learned how to do suctioning in nursing textbooks, it was done differently at the bedside. I did it as the nurse told me not to lose score” (P_6_). Another student said: “When a new patient was admitted to the ward, I was given responsibility. I had never encountered such a disease before, but the nurse expected me to do everything. My hands were shaking, but the nurse told me to do it at that moment to learn. I was under a lot of pressure” (P_16_). Routine-centeredness, no attention to patient-centered care, and inadequate student education reduce the quality of care.

Participants acknowledged that since they were supervised by a nurse and had to seek help from a nurse in case of complex problems, they were compelled to participate in caring for the supervisor nurse’s patients. Consequently, students endured a heavy workload. According to the regulations of the internship program, a nursing student, similar to the ward nurses, has the right to use the nurses’ resting room and other amenities. However, such facilities were not available for nursing internships in some hospitals. One of the nursing managers said: “We were not allowed to enter the nursing station. They said that we couldn’t sit on the chairs or rest in the resting room. We worked like nurses. We had the right to use the facilities” (P_11_). Inadequate rest, especially during the 12-hour night shift, can cause fatigue. Students’ drowsiness and fatigue can lead to errors and threats to patient safety.

## Discussion

This study aimed to reveal nursing students, newly graduated nurses, faculty, and nursing managers’ experiences implementing a nursing internship program to identify the strengths and weaknesses of the program for proper planning due to its novelty. In the present study, participants’ experiences were divided into five themes: ‘Facilitation of socialization process.’ ‘Filling the gap between theory and practice,’ ‘Improving self-confidence and independence,’ ‘Opportunity for clinical skill training,’ and ‘Achilles heel of the clinical setting.’

The students believed that the internship program, the opportunity to work with nurses, and teamwork strengthening, resulted in getting acquainted with professional rules and gaining a professional identity, thus forming a professional morale structure and greater adaptation to nursing, all of which resulted in student satisfaction. In the study by Ayaz et al. (2018), an internship program was conducted for senior nursing students, during which 3 h of theoretical and 18 h of practical training were held per week during one academic year. Students cared for patients for 15 to 20 days in each clinic under nurses’ supervision and discussed deficiencies with the clinic manager weekly. In this study, after the end of the course, students’ level of professional commitment increased [23]. In a study by Haleem et al. (2011) in which an internship program was conducted for 23 nursing students under the supervision of all ward nurses as well as the overall supervision of faculty members, a student survey at the end of the internship showed that it was a valuable experience, and students appreciated it. Students recommended that the course be continued and extended to other clinical courses [6]. In Hartwick et al.’s study (2010), in which an intensive internship program was conducted for newly graduated nurses, the program improved the student transition to a professional nurse; therefore, nurses’ retention in the profession increased, and new nurses’ intention to abandon in the first year of employment reduced [24]. According to our findings and previous studies, holding an internship program leads to an increase in the satisfaction and commitment of students and newly graduated nurses and plays a significant role in their viewpoint of nursing as a profession.

Participants’ experiences revealed that the internship program helped fill the theoretical and practical training gap. The faculty’s supervision and responses to students’ and nurses’ questions caused nurses to support and educate students about the necessary skills. Clinical knowledge and skills acquisition had distinguished nursing internship students; accordingly, supervisors were inclined to recruit students after graduation. The goal of education is learning, and every educator is gratified by achieving positive learning outcomes [2]. According to a study by Haleem et al. (2011), one of the main advantages of internship courses is that nurses are directly involved in student education, facilitating the learning process for nurses and students.

Moreover, discussions about patient care plans improve students’ learning and increase their enthusiasm for the clinical setting [6]. In Egypt, the implementation of the internship program was officially approved in September 2016 due to the importance of this course and newly graduated nurses’ inadequate experience and preparation. This course is supervised by instructors, during which teamwork is emphasized, and nurses are required to participate in 10 seminars [8]. In a study by Mollica et al. (2016), an internship program was held for students for 300 h in the oncology ward during the eight summer weeks. In this course, theoretical subjects were taught in lectures, and each student was assigned to an experienced oncology nurse as the main supervisor during their internship. The internship program effectively filled the gap between theory and practice and solidified students’ commitment [25]. Nash et al. (2018) used the internship program for six weeks (120 h) under the supervision of nurses to empower nursing students to provide post-operative care. Following the course, students’ awareness of and willingness to work in this field after graduation increased [26]. In the study by Roush et al. (2021), a 6-week internship program was held for nursing students. Initially, an 8-hour briefing meeting was conducted, including 4 h of lectures in the morning and 4 h of demonstration and practical training in the clinical skills laboratory. During the course, sessions were held for students to share their experiences, and interdisciplinary sessions were held for 4 h each week for further interaction. The first internship program resulted in 96% of NCLEX-RN students passing the course. In addition, 82% of hospital attendees were employed, and $ 216,993 were saved. Nurses who had not completed the internship program and entered the profession needed 12 to 16 weeks of briefing sessions, whereas those who completed the course needed only 10.5 weeks [27]. In Aboshaiqah et al.’s study, the internship program was conducted for newly graduated nurses for one year. Participants stated that nurses’ role in creating a sense of self-confidence in them to act as independent nurses was extremely effective. They also believed encouraging the nurses would increase this feeling and enhance their clinical competence [28]. According to our findings and previous studies, the internship program fills the gap between theory and practice, which in turn leads to the acceleration of the recruitment of internship students in hospitals after graduation.

Increasing students’ self-confidence was one of the significant features of the internship program. Students believed they had full responsibility for the patient, and nurses fully trusted them and considered them not students but nurses. In a study by Glynn et al. (2013), in which an internship program was conducted for graduate nurses, participants reported that their independence and self-confidence increased since they fulfilled activities independently. They also had the feeling of managing patients independently and appreciated the program implementation [5]. In the present study, working in different shifts and wards during the internship program led to exposure to various nursing procedures and acquiring skills. Kannappan et al.’s study (2020) found that the internship program could help nursing students improve their skills and clinical competence [9]. In this regard, in the study by Jokhio et al., the internship program was reported to be essential for achieving professional growth and acquiring skills [29]. Likewise, according to the study by Haleem et al. (2011), during this course, students’ critical thinking and patient care management improved [6]. According to the findings and previous studies, internship programs increase independence, self-confidence, clinical competence, and professional growth.

According to the findings of the present study, the “*Achilles heel of the clinical setting”* was the main challenge of the internship program. The present study revealed that the internship students were conflicted in the hospital because the care measures they learned differed from what was performed by the nurses in the hospital and caused them confusion. For the preceptors to become good role models for the students, the participants acknowledged that the faculty should examine their scientific and clinical competency before selecting them. Furthermore, most of the clinical education was conducted by preceptors, and the internship students stated that they felt abandoned in the clinical environment due to separation from the faculty. To solve this challenge, holding nursing rounds in the presence of faculty and clinical nurses will be helpful since the nursing internship needs the faculty’s study and guidance to prepare for the nursing round. Additionally, increasing supervisory sessions by faculty can help students reduce feelings of abandonment.

According to the participants’ experiences, abusing students to be involved in other patients’ care was among the problems of the internship program, and this finding was consistent with other studies [29]. To avoid such problems, in the study by Haleem et al. (2011), faculty members discussed the program with nurses before the internship program and exchanged phone numbers in case of a problem [6]. In the study by Uche et al., it was recommended that observers schedule regular one- or two-hour meetings with internship nursing students at the beginning of the program to discuss their progress and the problems they may encounter [8].

### Limitation

This study used the purposive sampling method, which can limit the transferability of the results. However, researchers attempted to use maximum variation by considering the participants’ characteristics regarding age, gender, and different stakeholders including faculty, nursing manager, newly graduated nurses, nursing internship student, and educators’ work experience, and a proportional number of participants for qualitative studies were used.

## Conclusion

This qualitative study was conducted for the first time to evaluate the internship program from the perspective of different stakeholders (nursing students, newly graduated nurses, nursing managers, and faculty). The results of the present study revealed that the implementation of an internship program for final-year nursing students played a role in preparing them for better professional performance, enhancing clinical skills, increasing self-confidence and independence, inspiring the nursing profession, strengthening professional commitment, and improving the chances of employment after graduation. In order to alleviate the identified challenges of the internship program, holding a briefing meeting with managers, supervisors, and faculty with the aim of determining working hours, performance standards, and amenities, such as lunch, dinner, and resting place, is efficient. Enactment of internship rules precludes professional regulations from being disregarded by hospitals, faculties, and students. It also prevents student abuse. Moreover, holding an introductory meeting with students will identify and introduce goals and expectations and help provide safe and quality care in the future.

## Data Availability

The data supporting the findings of this study are available on request from the corresponding author. The data are not publicly available due to privacy or ethical restrictions.
